# Improvement of pressure ulcer prevention care in private for-profit residential care homes: an action research study

**DOI:** 10.1186/s12877-016-0361-8

**Published:** 2016-11-25

**Authors:** Enid WY Kwong, Maria SY Hung, Kevin Woo

**Affiliations:** School of Nursing, Tung Wah College, Wylies Road, Kowloon, Hong Kong, China

**Keywords:** Action research, Pressure ulcer prevention, Residential care homes, Gerontology

## Abstract

**Background:**

A need exits to develop a protocol for preventing pressure ulcers (PUs) in private for-profit nursing homes in Hong Kong, where the incidence of PUs is relatively high and which have high proportion of non-professional care staff. The implementation of such protocol would involve changes in the practice of care, likely evoking feelings of fear and uncertainty that may become a barrier to staff adherence. We thus adopted the Systems Model of Action Research in this study to manage the process of change for improving PU prevention care and to develop a pressure ulcer prevention protocol for private for-profit nursing homes.

**Methods:**

A total of 474 residents and care staff who were health workers, personal care workers, and/or nurses from four private, for-profit nursing homes in Hong Kong participated in this study. Three cyclic stages and steps, namely, unfreezing (planning), changing (action), and refreezing (results) were carried out. During each cycle, focus group interviews, field observations of the care staff’s practices and inspections of the skin of the residents for pressure ulcers were conducted to evaluate the implementation of the protocol. Qualitative content analysis was adopted to analyse the data. The data and methodological triangulation used in this study increased the credibility and validity of the results.

**Results:**

The following nine themes emerged from this study: prevention practices after the occurrence of PUs, the improper use of pressure ulcer prevention materials, non-compliance with several prevention practices, improper prevention practices, the perception that the preventive care was being performed correctly, inadequate readiness to use the risk assessment tool, an undesirable environment, the supplying of unfavorable resources, and various management styles in the homes with or without nurses. At the end of the third cycle, the changes that were identified included improved compliance with the revised risk assessment method, the timely and appropriate use of PU prevention materials, the empowering of staff to improve the quality of PU care, and improved home management.

**Conclusion:**

Through the action research approach, the care staff were empowered and their PU prevention care practices had improved, which contributed to the decreased incidence of pressure ulcers. A PU prevention protocol that was accepted by the staff was finally developed as the standard of care for such homes.

**Electronic supplementary material:**

The online version of this article (doi:10.1186/s12877-016-0361-8) contains supplementary material, which is available to authorized users.

## Background

### Long-term residential care for Hong Kong’s older people

An aging world has led to a focus on long-term residential care for older people in health and social policies and planning. Long-term residential care homes (RCHs) are considered the last resort for older people. In Hong Kong, there are two main types of RCHs: government-subsidized or private. The RCHs run by the private sector are for-profit homes. The government-subsidized RCHs, which are operated by non-governmental organizations (NGOs), consist of two major types: care and attention homes and nursing homes (NHs). The government-subsidized homes are set up for care of older people according to level of dependence and required care of residents. Care and attention homes are eligible for older people who require moderate assistance in their daily activities while nursing homes are offered to those who need major assistance and have more care needs. The private for-profit RCHs, however, are not similarly classified and they are all named for-profit nursing homes in Hong Kong. The current demand for government-subsidized RCHs in Hong Kong is much greater than the supply. Consequently, a large number of older people have turned to for-profit private NHs [1].

In 2014, 69% of total residential care places for older people were in the private sector, while 31% were in government-subsidized RCHs [2]. In view of the increasing number of older people and the lack of planning to ensure that there will be an adequate supply of government-subsidized RCHs for older people in the future, the demand for for-profit private NHs will increase rapidly in Hong Kong. However, such homes have been criticized for providing substandard care [3], likely for the following charactistics. First, many potential residents in private NHs cannot afford to pay high fees. Therefore, to reduce costs in order to make a profit, private, for-profit NHs employ personal care workers (PCWs) as a large proportion of their care staff to perform the majority of personal care for residents. These PCWs are less educated, experienced, or trained, and also are paid a lower salary, than those in government-subsidized RCHs. Second, the resident-staff ratio (the number of residents cared for by each care staff member) is higher in private for-profit NHs than in government-subsidized RCHs. Third, many private, for-profit NHs employ health workers (HWs), who only have to complete a short training course approved by the Social Welfare Department, in place of professional nurses. Finally, since the minimum wage law took effect in May 2011, PCW have quit their jobs in favor of less physically demanding jobs. Therefore, these homes are being operated with less than three-quarters of the needed manpower [4], and the quality of their services has deteriorated.

### Pressure ulcers in RCHs

Pressure ulcers (PU) are one of the major clinical issues associated with service quality and patient safety. PUs occur in patients at all ages in various healthcare settings, but older people living in long-term RCHs are particularly vulnerable to developing PUs due to their frailty and multimorbidity in such settings. The prevalence of PUs in long-term RCHs varies in different countries, ranging from 4.5 to 27% in Italy [5], Ireland [6], the UK, the USA, Canada [7], Scandinavia, and Iceland [8]. The incidence ranges from 6.2 to 20.4% in the USA and Canada [7], Scandinavia, Iceland and Ireland [8]. In Hong Kong, the prevalence of PUs has been found to be as follows: in 16 randomly-selected, private, for-profit NHs in the Hong Kong East region it was 7.1% [9] and in three private, for-profit NHs in the Eastern District it was 25% [10]. By contrast, in two government-subsidized care and attention homes, the incidence was only 2.5% [11].

Prevention of PUs is a priority in RCHs. Pressure ulcers are not only an indicator of the quality of care in RCHs but also have a significant, negative impact on residents [12–14], care staff, and healthcare costs [15–17]. PUs have been rated the fifth most frequent cause of potentially avoidable hospitalizations [18]. In private, for-profit NHs in Hong Kong, PUs are one of the factors influencing the hospitalization of the residents [9].

### Pressure ulcer prevention

Pressure ulcer prevention is an area covered in the formal training that HWs and PCWs are required to undergo before and during their service. PCWs who are instructed and supervised by HWs or nurses if available perform daily personal care and other daily activities for residents to prevent pressure ulcers, such as repositioning, changing soiled incontinent products and skin care after each incontinence epidodes. However, the majority of private for-profit NHs do not have PU prevention protocols that are specially designed to guide non-professional care staff in preventing PUs.

### Previously designed PU prevention protocol for RCHs

Based on the practice guidelines from the European Pressure Ulcer Advisory Panel and the National Pressure Ulcer Advisory Panel [19], the current pressure ulcer prevention care being delivered in RCHs, and the roles and responsibilities of HWs and PCWs in RCHs in Hong Kong, the first author of this paper designed a pressure ulcer prevention protocol to guide HWs and PCWs in RCHs on how to prevent PUs. The protocol incorporates a PU risk assessment approach using the modified Braden scale, a flow chart to guide care staff on how to make correct decisions in performing appropriate preventive care, and a list of evidence-based prevention interventions. According to the flow chart, HWs and/or nurses are required to assess a patient’s risk of developing PU and to come up with a plan of care with preventive interventions customized for each resident according to the identified risk. PCWs are responsible for carrying out the planned interventions accordingly and for reporting any redness and lesions in the skin of each resident to HCWs and/or nurses for them to assess and confirm whether these are pressure ulcers. This protocol was previously pilot tested in two government-subsidized care and attention homes. The preliminary evaluation suggested that a reduction in pressure ulcers occurred after the implementation of the protocol [11]. In this preliminary study, data collected from focus group interviews with the care staff indicated that adherence to the pressure ulcer prevention protocol was inconsistent. Action research is considered a strategy for changing the practices of care staff and improving their adherence to the protocol.

### Action research approach to improving care

An action research approach has the potential to facilitate culture shift and practice change [20, 21]. The central tenant of this model is the importance to establish an equal partnership between action researchers and participants to understand, describe, interpret and explain the social context within which practice change and quality improvement is expected to occur. The action research process allows participants to identify the problems or issues, collect pertinent information, make decisions, plan for implementation, and involve in evaluation on an ongoing, cyclical basis. Through the research process, participants develop a sense of ownership of the problem and they are empowered to make the changes that are most relevant to practice. This research paradigm promotes a bottom up approach to change without imposition of values and practices [22].

In a systematic review of action research studies, emerging evidence supports this approach to promote acceptance of new care practices, continuation of new initiatives, and adoption of new projects in health care [23]. Other previous studies have also found that action research is a strategy to introduce evidence and change in practice [24], develop new practice [25], and improve the care practice [26] in healthcare settings. A recent review on implementing evidence-based nursing practice using action research concluded that the action research approach is promising, although there may have been publication bias [22].

An action research approach has been used in pressure ulcer prevention with favourable results. The project was implemented in St. Vincent’s Medical Center, and led to the development of the SKIN bundle (Surfaces, Keep the patients turning, Incontinence management and Nutrition) and to a reduction in the incidence of pressure ulcers. This SKIN bundle was shared with 67 acute hospitals of the Ascension Health organization [27]. In another study, Keen and Fletcher [28] demonstrated that a significant increase in the knowledge of staff on pressure ulcers and a reduction in the incidence of pressure ulcers occurred in a community hospital in Wales as a result of the development and implementation of a SKIN bundle through an action research strategy. In a private nursing home in Sunderland, the action research strategy allowed care staff to identify the barriers that they faced to providing quality PU prevention care. During the nine months of action research activities, the care staff reflected on and analyzed their preventive practices and developed and implemented PU prevention strategies. At the end of the study, the participants expressed increased levels of motivation and personal responsibility for preventing PU. There was an improvement in therapeutic relationships between care staff and residents and a reduction in the incidence of pressure ulcers [29].

Taking into account the relatively high incidence of pressure ulcers in private for-profit NHs in Hong Kong, the lack of a PU prevention protocol in many such homes, and the organizational charateristics discussed above, there is a need for private NHs to develop a PU prevention protcol to standardize the prevention care provided by non-professional care staff for improvement of the care. It is anticipated that the implementation of this protocol would lead to a shift in attitude and to changes the current pratices. Recognizing that change can evoke feelings of fear and uncertainty that may become a barrier to adherence, we adopted an action research approach in this study to introduce our team’s PU prevention protocol [11] to such nursing homes and to ensure staff participation, and incorporated their feedback in every step of the process of adopting and implementing the protocol.

### Study objectives

The objectives of our action research study were to:

1. explore how an action research approach can change the practices of care staff on the prevention of pressure ulcers for improving the outcome of care.
2. develop a pressure ulcer protocol for private for-profit NHs.

## Methods

### Study design

**Fig. 1:**
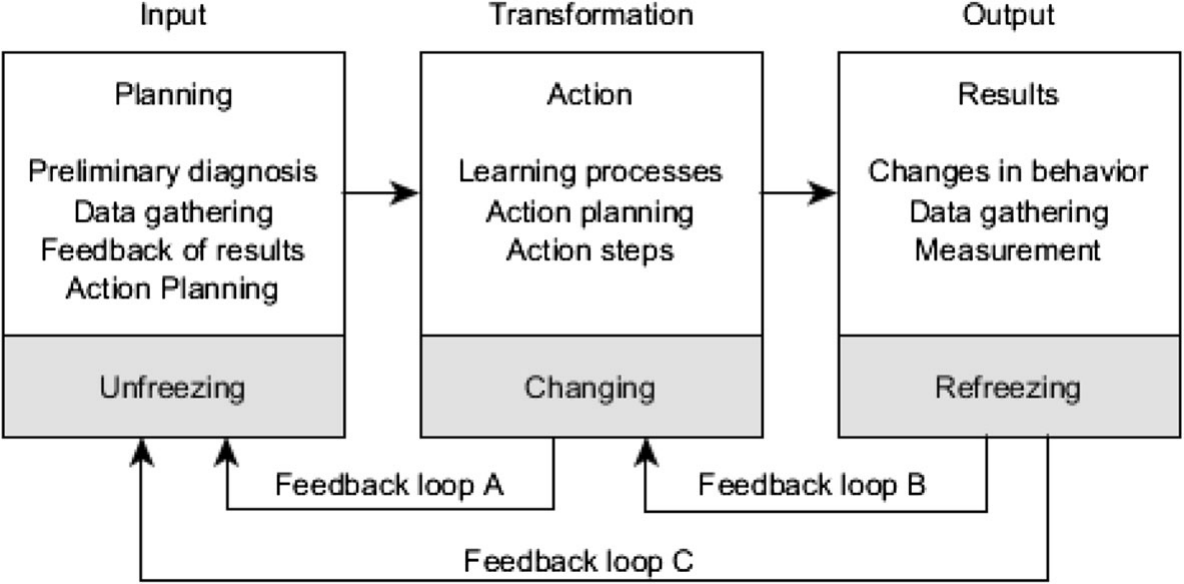
Systems Model of the Action Research Process (Lewin, [30]). Systems Model of the Action Research Process. The model is used to guide this study. It is an organizational development model of action research, with the aim of improving organizations through action research. There are three stages in this model: unfreezing, changing, and refreezing

The Systems Model of the Action Research Process [30] was adopted to guide this study. Lewin [30] first conceptualized this process and other behavioral scientists expanded on it. It is an organizational development model of action research, with the aim of improving organizations through action research. There are three stages in this model: unfreezing, changing, and refreezing. In the unfreezing stage, the people involved in the change become aware of change. In the changing stage, the situation is diagnosed and confirmed, and a new practice/model of behavior is explored. In refreezing stage, the new practice/model of behavior is evaluated. These three stages involve a cyclical process of change through three steps, namely, planning, action, and the results of action (Fig. 1).

#### Setting and sample

Before the commencement of the implementation of this protocol, four private, for-profit nursing homes in Hong Kong with a total of 474 residents perceived a need to improve their pressure ulcer prevention care and were willing to play an active role in the change. They therefore agreed to take part in this study. Newly admitted and existing residents, and care staff such as PCWs, HWs, and nurses (if available), were recruited as the study sample. Out of the four NHs, only two had nurses (NH-A had three nurses while NH-B had one nurse as the in-charge). All four NHs and their care staff voluntarily participated in the study. The total number of residents in the four NHs was similar in the three cycles of the implementation of the protocol: 474, 487, and 469, respectively.

#### Managing changes in the practice of the care staff

The three cyclical stages and steps in the Systems Model of the Action Research Process were adopted to manage the changes in the practice of the care staff on PU prevention in order to finally develop the PU prevention protocol.

### First cycle of unfreezing: planning

In this stage, there were some activities to come up with a preliminary diagnosis of the problem, gather data, and giving feedback on the results and the plan for action. The first author and the trained research assistant (RA) met several key people including the in-charge and experienced HWs or nurses selected by the in-charge of each NH to learn the issues relevant to pressure ulcers in their homes. They reported on the existing PUs (the prevalence) but did not have record of the incidence of PUs. The prevalence of PUs in these four homes ranged from 8 to 10%. Overall, the residence-staff ratio (the number of residents cared for by each care staff member) was around four to six. The turnover rate of care staff was acceptable, although this sometimes affected the care that was delivered. They agreed that the occurrence of PUs was an important issue in their homes, and efforts e.g., having a prevention protocol and training to care staff should be made to decrease them.

In each NH, the first author and the RA conducted four focus group interviews with various types of care staff (two with PCWs, one with HCWs, and one with nurses if there were nurses) to encourage them to share their current practices on pressure ulcer prevention care, as well as the barriers and difficulties that they faced, and their suggestions and expectations regarding the prevention of pressure ulcers in daily care. In addition, the PU prevention protocol, which had been previously designed and tested by the first author of this paper, was introduced to them in the interviews for their feedback [11]. The interview data were analyzed and interpreted for the action team to discuss and consider when planning the actions. The action team in each NH was formed. It consisted of four staff representatives from the PCWs, two from the HCWs, one from the nurses if applicable, the home in-charge, and the first author.

From the results of the interviews, the action team was informed that the care staff perceived their knowledge and skills on PU prevention to be inadequate, and that the manpower and resources for PU prevention were insufficient. With respect to comments regarding the PU prevention protocol, all staff agreed that there was a need to standardize the practice and they were willing to try the protocol through the action research approach. During the discussion, the staff expressed the concerns about workload. Specifically, they worried that pressure ulcer risk assessment was expected to be completed on a frequent basis and some preventive interventions were not currently being performed in the homes. Taking into consideration the constraints on manpower and resources in the NHs, and their current practices, the action team in each home decreased the frequency of assessing the risk of pressure ulcer developments and modified some preventive interventions in the protocol. In addition, the team acknowledge the learning needs of care staff and planned to design a training session on PU prevention to the care staff in each NH.

### First cycle of change: action

#### Training sessions to care staff

The data collected from the action teams of the four homes indicated that many care staff members, especially PCWs, were not aware of the importance of their daily care e.g., repositioning of the residents, use of positioning devices in pressure ulcer prevention. Besides, the majority of HWs did not use tools to assess the PU risk of the residents. Each nursing home’s action team designed a training session of 2.5 h for HWs and PCWs, and another training session of 1 h for HWs and/or nurses. The above mentioned knowledge deficit of the PCWs and HWs was emphasized in the training sessions.

Before implementing the proposed prevention protocol, the training session of 2.5 h was conducted by members of our research team for all types of care staff in each NH. The topics that were covered in the training session included the impact of daily personal care on PU prevention, skin assessments, pressure ulcer assessments, preventive interventions, the ways to use the PU prevention protocol and use of prevention materials/devices Another training session of 1 h was offered specifically to HWs and/or nurses to improve their skills in conducting PU risk assessments using the modified Braden scale [31–35] and in assessing and documenting PUs. In order to ensure that all types of staff were trained, the second and third rounds of the training sessions was provided to new care staff before the second and third implementation of the protocol. The inter-rater agreement among the HWs and/or nurses in the four NHs was between 95% and 98% for the modified Braden scale [31–35].

#### Implementation of the protocol

The care staff (PCWs, HWs, and/or nurses) in each NH carried out the protocol for six weeks according to the flow of the protocol. The HWs/or nurses assessed the PU risk of each resident using the modified Braden scale [31–35] and then planned the preventive interventions based on the identified risk. The PCWs delivered the care to the residents accordingly and inspected the residents’ skin when they performed perineal care and re-position the residents. If they noticed redness and/or lesions in the skin, the PCWs notified the HWs or nurses to assess whether they were PUs. During and after the implementation of the protocol in each cycle, some data were collected through various methods including field observations of prevention practices of care staff, focus group interviews with care staff, and assessments of the incidence of PU among the residents.

#### Field observations of PU prevention practices

Each NH was assigned a trained research assistant (RA) with a background in nursing to observe the day-to-day PU prevention practices of all types of care staff twice a week. The observations were conducted during different shifts and on different days, lasting for two hours each, without advance notification to the nursing homes. A validated observation form [10] listing some items associated with pressure ulcer prevention care was adopted to guide the observations. In the field observations, the RAs also focused on some areas that the care staff had brought up in the focus group interviews for the research team and the action team to clarify and/or validate. Before starting their observations, the RAs visited the NHs five times to familiarize themselves with the staff’s pressure ulcer prevention practices, build up a relationship with the care staff, and allow the care staff to get used to their presence when they were working.

#### Focus group interviews

Two focus group interviews involving five or six PCWs and three or four HWs in each NH were conducted by the first author and the RA using semi-structured guidelines at the end of each cycle in each home. One NH had three nurses, so they were placed in a single group for the interview. In each interview, the participants were facilitated in discussing their concerns, issues, difficulties, and suggestions regarding PU prevention care and the protocol. In addition, the care staff were asked about some areas that had been observed in the field observations, so that the research team and the action team could clarify and validate the data collected in the field observations. With the participants’ permission, the interviews were audio-taped and transcribed for analysis.

#### Assessing skin for the incidence and prevalence of PUs

According to the protocol, PCWs performed skin inspections once every day while providing personal care to residents, such as bathing them, turning their position, and providing the residents with perineal care to detect *new* ulcers on those who already had pressure ulcers and *first* pressure ulcers on those who did not have any at the beginning of the implementation of the first cycle. When the PCWs found redness or lesions in the skin, especially on the bony prominences, they reported them to the HWs or nurses and had them confirm whether these were pressure ulcers and assess their location, size, and stage. The PU incidence of each cycle in each home was summed up. Whenever the RAs went to the NHs for observations, they also inspected the skin of all of the residents to confirm the incidents of pressure ulcers that had been reported by the NHs and to also identify PUs that might have been overlooked by the NHs. In addition, the RAs assessed the PU prevalence of all residents in the homes just before commencement of the first cycle of implementing the protocol and just after the end of each cycle. The PU prevalence was recorded in the PU prevalence form. Training on how to assess the size, location, and stage of PUs was provided to the four RAs before the commencement of the study, and 95% agreement in assessment of PUs was obtained among the RAs.

### Data analysis

Qualitative content analysis [36] was adopted to analyze the data obtained from observations and focus group interviews. The software of NVIVO (version 10) was used. The data were independently coded by the first author and the trained RA. They then discussed the codes until they reached a consensus. They used the codes to further develop the sub-themes, which were then clustered into themes based on several discussions and on a consensus reached among the members of the research team. The incidence and prevalence of PUs were calculated and compared across time and across the four NHs studied.

### Study rigor

Data triangulation was adopted by collecting the data from different types of care staff (HWs, PCWs, and nurses) and residents in the NHs. Methodological triangulation was also employed, including focus group interviews, field observations, and assessments of the residents for the incidence and prevalence of PUs. Through the focus group interviews and field observations, the first author and the RAs in the nursing homes validated the data collected from these two sources. The data and methodological triangulation increased the credibility and validity of the results and also provided a more detailed picture of the care staff’s practices regarding PU prevention and changes to those practices. Further, member checks were performed before each action team meeting in each NH to validate the data that were collected and the results. The transcript of each focus group interview was given to the interviewees for clarifications, if any, and validation. A summary of the field observations was reported to the PCW and HW groups for their validation.

### First and second cycles of refreezing: results

Three cycles of planning, action (including the implementation of the strategies to address the issues that had been identified and the implementation of the protocol), and results were carried out in this action research study. In this article, the results in the first and second cycles were presented together.

Through the triangulation of data from the focus group interviews and/or field observations in the analysis, the themes were identified. The themes included prevention practices after the occurrence of PUs, the improper use of pressure ulcer prevention materials, non-compliance with several prevention practices, improper prevention practices, the perception that the preventive care was being performed correctly, inadequate readiness to use an assessment tool to assess the risk of developing PUs, an undesirable environment, the supplying of unfavorable resources, and various management styles in the homes with or without nurses. There were some sub-themes under each theme (Additional file 1). The themes and the sub-themes were considered issues that could potentially increase the risk of PU development in each home. Compared with the incidence of PUs in the first cycle of the implementation of the protocol, the incidence increased in two NHs (NH A and B) but decreased in another two NHs (NH C and D) in the second cycle. Compared with NH-B, the increase in the incidence of PUs in NH A with three nurses was greater (from 9.2 to 11.7%, an increase of around 27%) (Additional file 1: Table S3). The decrease in the incidence of PUs in NH-C and D was very low (from 8.8 to 7.2% in NH-C and from 4.9 to 4.4% in NH-D). However, an improvement of pressure ulcer incidence was seen in the four NHs from the first cycle to the completion of the third cycle.

### Second and third cycles of unfreezing: planning (feedback loop C)

After the data analysis in first and second cycles of the implementation of the protocol, the action team of each NH met to thoroughly discuss and interpret the issues (themes and sub-themes) that had been identified. The four action teams agreed that, along with the availability of resources, these issues were relevant to the knowledge and understanding, workload, work commitment, and empathy of the members of the care staff. Several issues also involved family members and doctors. After each action team engaged in negotiations and reached a consensus, strategies were planned to address these issues for implementation of the second and third cycles of the changing stage (action). Each in-charge announced the issues and the strategies to all of the care staff members in her home. In general, the care staff members in all four NHs agreed with the strategies, although there was minor modifications were made in two homes. The following strategies, which were planned and carried out before the second and/or third cycles, were presented together in this article.

### Second and third cycles of changing: action

Following feedback loop C, the planned strategies were carried out before commencement of the second and/or third cycle of the implementation of the protocol.

### Implementation of the strategies

#### In-service training

The research team conducted the training for all care staff in the four NHs to address the issues that were identified. The training reinforced the knowledge and also demonstrated the skills required by the care staff for prevention care. These included the concepts and importance of prevention, the proper use of pressure ulcer relieving materials, such as appropriate methods for checking the function of pressure relieving mattresses, applying heel protectors and placing pillows to support the positioning of the residents in order to minimize the pressure on their sacrum, the acromion process of the shoulder blade, and the trochanter of the femur, and the importance of conducting risk assessments of the development of PUs. They were also given an explanation of how some improper daily care procedures that had been identified increased the risk that the residents would develop PUs. They were also advised to pay more attention and effort to minimizing the risk posed by their improper care practices, including the inappropriate positioning of residents in beds, the overly tight application of physical restraints, the use of inner and outer napkins for incontinent residents, insufficiently frequent changes of napkins, prolonged sitting time for the residents, and not using pressure-relieving seating cushions. It was emphasized that physical restraint must be applied ethically. The ethical issues, principles and guidelines on use of restraint were explained in details. It was highlighted that use of physical restraints is the last resort. The care staff should consider some other strategies to maintain the safety of residents before applying physical restraint to residents and they were reminded of following the guidelines to apply physical restraint to residents. Besides, the care staff were clearly told that using inner and outer napkins to incontinent residents was inappropriate practice that increased the risk for PU development of residents and decreased their comfort. This issue was further discussed with care staff to have their consensus of the practice, that was presented below at Discussions with care staff.

The training emphasized the importance of several areas of PU prevention in which the care staff were not compliant. These included conducting daily skin inspections to identify redness, dryness, and lesions in skin in a timely manner, applying body lotions to bony prominences of limbs, re-applying heel protectors that had slipped off, and assessing the risk of developing PUs. In addition, a more detailed explanation was given to care staff, who mistakenly thought that it was correct to use inner and outer napkins and to allow chair-bound residents to sit out for lengthy periods, that these practices only served to decrease the comfort of the residents and to increase the risk of developing PUs.

The in-charge of each nursing home explained to the care staff that it was not possible to change the location of beds placed against a wall or partition, but that the care staff were strongly advised to find a co-worker to help them provide care to heavy and frail residents at the same side of the bed and also to use a lifting belt and a transfer slide for lifting and transferring procedures if necessary. More lifting and transferring materials were prepared, and the skills required to use these materials were demonstrated.

### Discussions with care staff

Meetings were organized in each home to allow the majority of care staff, the home in-charge, and the first author to discuss several issues, including pressure ulcer risk assessments, pressure ulcer prevention materials, the use of inner and outer napkins, the frequency with which napkins should be changed, lengthy periods for sitting out the residents, staff communication, and management styles. After thorough consideration, discussions and negotiations, it was agreed that the following actions would be carried out.

The participants in the meetings agreed to the following approach to simplifying the method and frequency of assessing risks. The HWs used items in the modified Braden scale to identify the risk factors for bed- or chair-bound residents who were not able to reposition themselves on their own. This identification was performed when the resident was admitted, once every three weeks, and after a significant change in the resident’s health status. The HWs or nurses planned preventive interventions to address the identified risk factors, instead of using the cut-off score to identify those at risk. The PU protocol was modified accordingly. Regarding the PU prevention materials, they agreed to take more initiative in approaching family caregivers to give them a more detailed explanation, before the occurrence of PUs, of the importance of the PU prevention materials for residents who were at risk of developing PUs. In addition, they agreed to approach doctors before the occurrence of PUs in order to draw their attention to the residents at risk of developing PUs and to recommend the purchase of these PU prevention materials. The in-charges of the NHs also notified the doctors of this issue.

The PCWs understood the disadvantages of using inner and outer napkins for incontinent residents, but they expressed concern about their heavy workload. Therefore, they used inner and outer napkins only during the night shift when manpower was less. In the daytime shift, they agreed to check the napkins more frequently and change them when necessary, and not wait until the routine napkin round. Regarding the lengthy sitting-out period for chair-bound residents, they were willing to move the residents from their beds to the chairs a bit later, around 30 min later, to shorten the sitting-out time. Two in-charges in the NHs with nurses accepted the suggestion that two seat cushions be purchased for chair-bound residents.

In terms of management style, the care staff were clearly reminded of the roles and responsibilities of PCWs, HCWs, and nurses (if available), and supervisory roles for HWs and nurses were emphasized. The importance of handing over the cases and the care required in each shift was also explained. The care staff members were provided with an opportunity to freely share their thoughts on the barriers to and expectations for reporting on the cases, the care required, and the supervision. After this, each home came up with its preferred ways of supporting, monitoring, and supervising different types of care staff, and of handing over cases between the care staff.

### The purchase of materials for PU prevention

Two of the nursing homes (Homes A and B) bought two pressure-relieving seat cushions for each of their chair-bound residents to use on a trial basis, but the other two nursing homes did not. All four homes agreed to ask members of the residents’ family to buy them for their chair-bound residents. In addition, the four homes increased the number of the lifting belts and transfer slides for care staff to use.

### Management of the residents’ possessions on the beds

It was not at all easy to manage the clutter on the residents’ beds. Due to insufficient space, it was impossible to give additional bedside cabinets to each resident. The in-charge of each nursing home explained to the family members how these belongings, which occupied much of the bed, affected the care provided to the residents. The family members were strongly advised to avoid buying unnecessary items for the residents. In the meantime, the PCWs cleared and tidied the beds every day and the existing bedside cabinets at least once a week in order to create more space to store as many of the belongings that had been placed on the beds as possible.

### Second and third cycles of the implementation of the protocol

After the above strategies were implemented, the second and third cycles of the implementation of the protocol were carried out in the same way as the first cycle (each cycle of implementation lasted for six weeks).

#### Third cycle of refreezing: output

After the third cycle of the implementation of the protocol and the collecting of data, the data from the focus group interviews and field observations were triangulated in the analysis in the same way as in the previous two cycles. We put a stop to the action research cycle after completion of the third cycle. This was because it was observed that the care staff were willing to follow the protocol to carry out the prevention practices andpositive changes in the practices of the care staff (Additional file 1) were identified, and the PU incidence of the residents was noted (Additional file 1: Table S3), although several areas needed to be further managed.

The pressure ulcer prevention practices of the care staff improved after the first cycle. At the third cycle, the majority of the HWs and all nurses complied with assessing the chair- and bed-bound residents using the items of the modified Braden scale. In providing preventive care, the PCWs were able to use the pressure ulcer prevention materials, including heel protectors, pressure-relieving mattresses, and seat cushions, appropriately and in a timely manner. The prevention materials were applied on some residents who had not developed PUs but who had high risk of PU development. Overall, the PCWs were empowered in the areas of skin care, the positioning of the residents, incontinence care, and the use of physical restraints to improve the quality of the care that was delivered. In home management, communication among the care staff on the caring of residents had been enhanced and supervision of PCWs had improved in all four homes. The homes without nurses had developed a formal way to hand over the cases and required care in each duty shift. It was also observed that with the efforts of the PCWs, the clutter on the majority of the residents’ beds had decreased.

Due to positive changes in the practices of the care staff in pressure ulcer prevention care, it is not surprising to see a positive outcome in care, namely, a decrease in the incidence of PUs in the four NHs. The incidence of PUs in the four NHs ranged from 9.2 to 3.4% in the period of the first cycle of the implementation and then dropped to 5.4 to 1.7% in the period of the third cycle of implementation. Overall, the incidence decreased in each NH between the first and third cycles of the implementation of the protocol. In NH-A and NH-B (with nurses), the decrease in the incidence was 64% (dropping from 9.2 to 3.3%) and 50% (dropping from 3.4 to 1.7%) respectively. In NH-C and NH-D, the decrease was less dramatic, at 39% (dropping from 8.8 to 5.4%) and 8% (dropping from 4.9 to 4.5%), respectively (Additional file 1: Table S3). The staff and resident ratio (the number of residents cared for by each frontline staff member) in the four NHs was similar throughout the whole study.

## Discussion

### Development of the PU prevention protocol

This study provided evidence that three cycles of unfreezing (planning), changing (action), and refreezing (results) stages in the Systems Model of Action Research Process [30] changed care staff’s practice in pressure ulcer prevention and finally developed the PU prevention protocol. The care staff members in the four NHs were empowered through their involvement in the cyclical implementation of the prevention protocol, self-reviews of their own practice, the identification of some preventive care issues and barriers that potentially lead to the development of PUs, the planning and implementation of strategies to address these issues/barriers, and the evaluation of the protocol. This action research approach was workable as seen by the care staff’s willingness to follow the protocol to carry out prevention care and in the positive changes in their pressure ulcer prevention practices, which resulted in a decrease in the incidence of PUs in the four NHs in the third action research cycle.

### Sustainability of the PU prevention protocol

The evidence-based prevention protocol was developed in this action research study. We need to pay great attention to its sustainability, as stated in the systematic review of pressure ulcer prevention programs [37]. Through field observations and the focus group interviews, it was determined that the care staff members accepted this protocol and were willing to carry it out in their homes during this study. However, in order to ensure the sustainability of this protocol after the completion of this action research study, some relevant major issues need to be addressed, for example, the manpower and resources of the NHs, the staff’s knowledge and skills on PU prevention, and the support and supervision of the care staff. We strongly suggest that the NHs continuously take a bottom-up approach to getting frontline care staff involved in regularly finding care issues and barriers relevant to the implementation of this protocol, planning the strategies to address those issues and barriers, and implementing and evaluating the agreed-upon strategies afterwards. Giving the care staff a sense of ownership of the care guided by the protocol is crucial if they are to adhere to it properly and cooperatively.

### Preventive care issues identified

The preventive care issues that were identified are interrelated. They included prevention after the occurrence of PUs, the improper use of pressure ulcer prevention materials, non-compliance with some prevention practices, improper presssure ulcer prevention practices, the perception that the preventive care is being performed correctly, inadequate readiness to adopt an assessment tool to assess the risk of developing PUs, the supplying of unfavorable resources, and various management styles in the NHs with or without nurses.

In the provision of care, the care staff did not provide preventive care materials, including heel protectors and pressure-relieving mattresses, until they found that pressure ulcers had developed. This practice was likely caused by their knowledge deficit and wrong concept of PU prevention, the unfavorable supply of PU prevention materials (e.g., heel protectors and pressure-relieving mattresses), or even the complete unavailability of PU prevention materials (e.g., pressure-relieving seat cushions). Family members were also not willing to spend money on the prevention materials if PUs had not been observed because they might have thought that the materials were being used for treatment instead of prevention, or that the PUs were not serious enough for these materials to be used. In addition, due to the healthcare system, a doctor’s referral to purchase prevention materials is accepted only for residents who are recipients of government social security allowances.

In this study, improper practices were observed among the care staff members in the four NHs. These included failing to adjust a heel protector that has slipped off, the use of inner and outer napkins, lengthy sitting-out times for chair-bound residents without the provision of a pressure ulcer seat cushion, the use of inapproprate methods and inadquate frequency to change napkins, causing the wetbuttocks of the residents, the inappropriate positioning of bed-bound or chair-bound residents, the use of an air-ring or non-pressure relieving seat cushion for chair-bound residents, and the tight application of physical restraints. All of these improper prevention practices increase the risk to residents of developing PUs, especially those who are chair-bound and bed-bound. The knowledge deficit of the care staff and an unfavorable supply of resources including prevention materials and/or manpower and inadeqaute staff supervision are possible reasons for the improper delivery of prevention care to residents in nursing homes. Apart from improper preventive pratices, the care staff did not regularly inspect the skin of the residents to identify dryness and redness/lesions, and did not apply body lotion to the dry skin on the bony prominences. Inadequate manpower and a knowledge deficit might also be the reasons for their non-compliance with these practices.

With regard to the improper use of pressure ulcer materials, some pressure ulcer relieving mattresses were not adequately inflated, pillows were not appropriately placed to support the positioning of some residents to decrease the pressure on their bony prominences, and heel protectors were applied tightly. As with the performance of improper prevention practices, all of these are possibly due to a deficit in the knowledge and skills of the care staff, inadeqaute manpower, and ineffective staff supervision.

Assessing the risk of developing PUs is the first step in the practice of preventing PUs. An adequate assessment allows us to timely identify residents at risk of developing PUs and to perform appropriate and timely PU prevention care. However, the care staff did not display sufficient readiness to adopt the modified Braden scale to assess the PU development risk of the residents. They were reluctant to perform this task at the first cycle because they found it to be time-consuming and unnecessary. In sufficient manpower also made it difficult for them to perform the task frequently on the residents. From their work experience and knowledge, they knew that bed- and chair-bound residents who were unable to reposition themselves on their own were at a high risk of developing PUs so they thought it was not necessary to use a scale to assess the risk. Their views on using the assessment scale were also similar to those of the care staff in a local study involving two government-subsidized NHs, in which it was reported that the care staff were not compliant in using the scale to identify the risk of developing PUs because they thought that they could assess the risk based on their work experience and professional judgment [10, 11]. In addition to the use of professional judgment to assess risk, it is necessary to have a simple, effective, and user-friendly tool to guide the care staff in their assessments of risk, especially in private for-profit NHs where the majority of the care staff are not professionals and have received less training than those in government-subsidized homes.

The environmental aspect included the tendency to put many of the residents’ belongings on their beds and having one side of the bed against a wall or partition. This likely decreased the quality of the care staff’s bedside care, as it affected their ability to turn the residents, change their position, and transfer the residents, resulting in an increased risk of the residents developing PUs, such as through shearing force and friction. This environmental aspect is always neglected as a barrier to the prevention of PUs in for-profit NHs in Hong Kong.

Different management styles in homes with or without nurses were identified, including in the areas of staff supervision and in the handing over of cases in each duty shift. Effective staff supervision allows nurses and HWs to sufficiently support, monitor, and evaluate the work of PCWs, which likely results in their compliance with proper preventive pracctices in NH settings. Besides, the care staff would understand the health condition of the residents, the rationale for the care delivered to the residents, and the specific tasks that need to be performed for the residents if there is a formal way to hand over the cases for all care staff in each duty shift. However, in the nursing homes without nurses, HWs did not actively supervise the PCWs and there were no formal procedures for handing over the cases in each duty shift. The PCWs were only told what needed to be provided to specific residents if the necessity arose. This may explain why the private for-profit nursing homes without nurses had a lower percentage of decrease in PU incidence than in the NHs with nurses in this study. This is consistent with the findings in Kwong et al.’s study [10] that not having nurses is one of the risk factors in the development of PUs in private for-profit NHs.

Through triangluation of the data obtained from the focus group interviews and field observations, the consistency of the preventive issues was confirmed. The exception was several issues that were observed through the field observations but were not mentioned by the care staff members in the focus group interviews after the completion of the first cycle. Those issues are improper or non-compliant preventive practices, including the overly tight application of heel protectors and limb restrainers, skin inspections, the identification of redness of the skin, the appropriate use of pillows to support the positioning of the residents, the fixing of heel protectors that have slipped off. All these care issues increased the risk of PU development [38]. Some of the issues that were identified in this study are same as those that were reported as barriers to PU prevention care in Kennedy’s action research study [29]. In the focus group interviews conducted after the completion of the second and third cycle, the care staff members responded that they were paying more attention to those care practices and were making improvements in those areas. The improvement on these issues was also observed through the field observations.

To conclude the above discussion, if care staff in Hong Kong private for-profit NHs are to comply with the proper practices for preventing the development of PUs, they need to have sufficient knowledge and skills to do so, to be supplied with sufficient resources including prevention materials and manpower, and to have effective supervision. Apart from these criteria, we cannot ignore the importance of staff members mentality, their commitment to the job and the value that they place on the job, and their empathy when delivering quality care including pressure ulcer prevention care although these issues were not explored in this study.

### Changes in staff practices

Through a self-review of their practices, peer influence, in-service training, discussions, and negotiations, the care staff accepted the prevention protocol to guide their PU prevention care and were also empowered to make some positive changes to their practices.

#### Staff compliance with risk assessments and skin inspections

When the method and frequency of assessing the residents’ risk of developing PUs were modified, the care staff became more compliant. Assessing risk is the first step in the effective prevention of pressure ulcers. Valid and reliable PU risk assessment scales are underused [39]. Indeed, using assessment tools is not the only means of identifying the risk of developing PUs. This study has found that, when planning interventions to minimize the risk factors involved, using the risk factors from a reliable and valid tool, together with the knowledge, judgment, and experience of care staff, is also an effective approach to assessing risks. For effective management, it is important to achieve a good balance between the complexity of a care task and staff compliance with that task. Involving the care staff in planning care tasks is highly recommended as a strategy to achieve this balance.

The PCWs expended more effort in inspecting the skin of the residents during their provision of perineal care and in repositioning the residents. They also became more aware of the redness, breakdown, and dryness of the residents’ skin. They reported any redness and breakdown of skin to the HWs or nurses to manage, although they might not have known whether or not they were PUs. Compared with two previous local studies, which reported that PCWs failed to identify and report redness in the skin of residents [10, 11], the quality of the PCWs’ practice in this aspect was better. This change is important because PCWs are the crucial team in frontline care at either government-subsidized RCHs or private for-profit NHs. Their timely detection of PUs results in the timely management to minimize the deterioration of pressure ulcers, and in the suffering and pain of the residents which are very important in residents’ quality of life. In addition, PCWs identified dry skin, especially on the residents’ bony prominences, and apply body lotion accordingly. This practice allows the skin to retain its moisture, thereby preventing PUs.

#### Use of pressure ulcer prevention materials

Care staff members were found to use the prevention materials in a proper and timely manner. These included using heel protectors, pressure-relieving mattresses, and pillows. Residents who were at a risk of developing PUs but had not done so were also given the prevention materials. This change implies that the care staff had improved their concept and knowledge of prevention, and also that the supply of these materials (especially heel protectors and pressure ulcer relieving mattresses) had increased. Together with their proper performance of prevention practices, the risk that the residents would develop PUs decreased.

#### Pressure ulcer prevention practices

The PCWs improved their prevention practices in the areas of skin inspections, the positioning of the residents, the use of napkins, and the use of physical restraints. They paid more attention and effort to the proper positioning of the residents, with the proper use of pillows to support their position in bed, which is an effective method for preventing the development of PUs in residents, especially the bed-ridden.

From the point of view of the care staff, using inner and outer napkins for incontinent residents enabled them to decrease the frequency with which they changed the napkins. They did not feel or see the wetness of the inner napkin, as it was covered by the outer napkin, so they did not change both the inner and outer napkins. However, this practice increased the wetness of the skin on the residents’ buttocks. Following the strategy that was agreed upon to improve this inappropriate practice, the care staff used one inner and one outer napkin for each incontinent resident only on the night shift, because there was less manpower at night, and also checked the dryness of the napkins before the residents’ meal times, apart from the scheduled twice-per-shift checking and changing of napkins. With this change, the number of residents with wet napkins and buttocks decreased. It helped to minimize their risk of developing PUs. However, it was observed that some residents still had wet napkins and buttocks.

Safety vests and hand restraints were appropriately applied to the residents to allow them sufficient space to move on their own in bed. This not only gave them comfort but also reduced the duration of pressure on their bodies, and thus decreased their risk of developing PUs.

### Change in home management

The PCWs gradually experienced an improvement in supervision. Through effective supervision, the PCWs were more cooperative at work and more willing to accept advice and instructions from the HWs and/or nurses. All four homes had a formal handing-over time to allow all care staff on duty to report and learn about the health conditions of the residents and the tasks that needed to be performed for the residents in each duty shift. Communication on the care of the residents was also enhanced, especially among the care staff in the homes without nurses. Previous studies identified communication and coordination as important predictors of staff cohesion [40, 41], which was negatively associated with the development of PUs in a study involving nursing homes [42].

### Change in environment

The environmental issue that had changed was the number of belongings left on the residents’ beds. These decreased after the care staff explained the importance of reducing clutter to the family caregivers of the residents and made an effort to tidy up the beds regularly. In addition to comfort, the residents had more space to move around in bed, and the care staff members were able to more effectively perform bedside care.

It is impossible to change the location of a bed in which one side of the bed is in contact with the wall or partition in the NHs because it allows more beds to fit into the homes, resulting in the use of less space and greater profits. As the NHs were not spacious enough for the use of a device (e.g., hoist) to pick up the needy residents and transfer them from their beds to chairs, this created a barrier to the provision of good quality bedside care for the prevention of PUs and also to the occupational safety of the care staff. To adapt the unchanged bed location, it was observed that one or two care staff member(s) standing on the same side of the bed used the lifting belts or transfer slides to lift and transfer several residents who were weak and heavy. However, due to the insufficient number of belts and slides placed in each room, and the insufficient number of staff, it was still the case that only one staff member at one bedside who did not use the belt and/or the slide lifted and/or transferred the weak residents. This practice increased the friction and shearing force on the residents.

### Changes in PU development

An increase in the incidence of PUs was found in Home A and Home B (with nurses), while a slight decrease was seen in Home C and Home D (without nurses) at the second cycle. The increase in the incidence of PUs in the Home A and Home B might had been caused by the relatively higher staff turnover rate there during the second cycles. This increase in the incidence of PUs might have drawn the attention of the staff in these two homes. The nurses there started to pay more attention and make more efforts to observe, monitor, and supervise their staff members (HWs and PCWs), resulting in a gradual change in their practices. The positive changes in the practices of all care staff in the four NHs most likely led to a decrease in the incidence of PUs by the end of the third cycle. The decrease in PU incidence in Homes A and B was greater than that in Homes C and D. Perhaps this was because Homes A and B had nurses to supervise and monitor the PCWs and HWs in the prevention of PUs and also because the ratio of residents to care staff is comparatively lower in Homes A and B as reported in Kwong et al.’s study [10]. There was no obvious improvement in the prevalence of PUs in any of the four NHs after the implementation of the protocol, perhaps because some residents had developed their PUs when they were hospitalized (hospital-acquired PUs).

### Some issues that need to be addressed

The inadequate supply of pressure ulcer prevention materials, including pressure-relieving seat cushions and mattresses and heel protectors, is an issue that has yet to be resolved. Preventive materials have still not been used on some residents with PUs or who are at risk of developing PUs. The sitting-out time for frail residents is relatively long (around 2–3 h), but there are not enough care staff to change the residents’ position for pressure relief more frequently and an insufficient number of pressure-relieving seat cushions have been supplied, or none at all. Inner and outer napkins are not being used during the day and the frequency with which napkins are checked and changed has increased, but some residents have still been found to have wet napkins and buttocks. This implies that the change has not been effective enough for all incontinent residents. The inadequate manpower is a barrier to improving the quality of PU prevention care [29] in the two areas that are long sitting-out time and use of inner and outer napkins in night shift. Finally, the issue of the location of beds is one that is difficult to change so some strategies e.g., availability of a set of lifting belt and transfer slide to be placed in each room should be further considered to engage staff members who stand in one bedside in performing lifting and transferring procedures. All of these important issues require continued attention and effort from the owners and care staff of private, for-profit RCHs in Hong Kong and in other countries where the situation might be similar.

## Conclusion

This study has provided evidence that an action research approach is effective at changing the pressure ulcer prevention practices of the care staff in the four participating NHs. An evidence-based pressure ulcer prevention protocol acceptable to the staff was also developed for private, for-profit RCHs where the majority of the care staff are not professionals and where achieving a balance between making profits and delivering quality care is a major challenge. For the protocol to be sustainable in the NHs after the completion of the action research study, it is recommended that a bottom-up approach be followed, where frontline staff are encouraged to be continuously involved in regularly identifying barriers to the implementation of the protocol, planning the strategies to address these barriers, and implementing and evaluating the strategies. Through the triangulation of the data in this action research study, the major issues and barriers that directly and indirectly increase the risk to patients of developing PUs and some positive changes in the pressure ulcer prevention practices of the care staff in the last action research cycle were identified. These changes most likely led to the decrease in the incidence of PUs in the NHs. It is recommended that further experimental studies are conducted to test the effect of this finalized protocol.

## Additional file

Additional file 1: Table S1. Themes and sub-themes were identified through triangulation of the data from the focus group interviews and field observation. These themes are well-supported from the focus group interview data and/or observation data. They were the care issues required to manage in the action phase. **Table S2.** Themes and sub-themes identified were the changes of the care staff in their daily pressure ulcer prevention practice. **Table S3.** The incidence and prevalence of pressure ulcers in the three cycles of implementation. (DOCX 47 kb)

## Abbreviations

HW: Health workers
MBS: Modified Braden Scale
NHs: Nursing homes
PCWs: Personal care workers
PU: Pressure ulcer
RA: Research assistant
RCHs: Residential care homes
