# Supplementary material for: Improvement of pressure ulcer prevention care in private for-profit residential care homes: an action research study
Source: BMC Geriatr. 2016 Nov 25;16:192. doi: 10.1186/s12877-016-0361-8 (PMC5123273; doi:10.1186/s12877-016-0361-8)
Supplement: Additional file 1: Table S1. — Themes and sub-themes were identified through triangulation of the data from the focus group interviews and field observation. These themes are well-supported from the focus group interview data and/or observation data. They were the care issues required to manage in the action phase. Table S2. Themes and sub-themes identified were the changes of the care staff in their daily pressure ulcer prevention practice. Table S3. The incidence and prevalence of pressure ulcers in the three cycles of implementation. (DOCX 47 kb) [file 12877_2016_361_MOESM1_ESM.docx]

Table S1. Issues identified through the triangulation of the data from the focus group interviews and field observations

|  |  | | |  | | | **Focus Group Interviews** | | **Field Observations** | |  |
| --- | --- | --- | --- | --- | --- | --- | --- | --- | --- | --- | --- |
|  | Themes | | | Sub-themes | | | Verbatim | | Data observed | |  |
| 1 | Prevention after occurrence of PUs | | | 1.1 Consideration not given by staff to using PU prevention materials until the occurrence of PUs | | | After finding PUs on the residents’ heels, we started to think about giving heel protectors to them and asked the doctors to assess whether heel protectors were needed.… If we think about giving heel protectors to residents before they have PUs, many residents may need them. (PCWs and HWs) | |  | |  |
|  |  |  |  | 1.2 PU materials not bought by family members until the occurrence of PUs | | | Most family members do not buy heel protectors and/or pressure-relieving mattresses for residents or rent the spare materials from our home if they do not see pressure ulcers on them. (PCWs and HWs) | |  | |  |
|  |  |  |  | 1.3 Referrals by doctors to purchase PU prevention materials not made until the occurrence of PUs. | | | These prevention materials for residents who are receiving the comprehensive social secruity allowance can be funded by the government if the residents have a doctor’s referral to purchase them. However, doctors usually only make referrals for cases where pressure ulcers have already developed. (PCWs and HWs) | |  | |  |
|  |  | | | 1.4 Pressure ulcer prevention materials not to be used by residents without pressure ulcers | | |  | | - Some weak and frail residents who did not have pressure ulcers but who were at risk of developing them were not offered pressure-relieving mattresses, heel protectors, or press-relieving seat cushions.  - One heel protector was applied on some residents with pressure ulcers. | |  |
| 2 | Improper use of pressure ulcer prevention materials | | | 2.1 Limited skill in  checking the function of pressure relieving mattresses | | | When I see that the buttocks area of a mattress is intruded, I know that the mattress is deflated and perhaps leaking air…. This is the only method I use to check the mattress.… I am not sure other methods can be used. (PCWs) | |  | |  |
|  |  | | | 2.2 Pressure relieving  mattresses not adequately functional | | |  | | - Some mattresses were deflated.  - Although some mattresses were  turned on, some segments of the  mattress did not inflate. | |  |
|  |  | | | 2.3 Tight heel protectors | | |  | | - Heel protectors were applied tightly. | |  |
|  |  | | | 2.4 Pillows not placed correctly to support the positioning of the residents | | |  | | - Pillows were laid flat at the side of the bed and next to the residents only.  - The pillows were used to support the residents in a lateral position, but the residents slept laterally at either more than 30 degrees or less than 10 degrees.  - The bony prominences of the residents’ sacral area at the lateral position were in contact with the mattresses. | |  |
| 3 | Non-compliance with prevention practices | | | 3.1 No daily inspection of residents’ skin during daily personal care. | | |  | | - The residents’ skin was not inspected daily by the care staff members when they performed personal care for the residents, e.g., changing their position, providing perineal care, changing their napkins, and giving them showers. | |  |
|  |  | | | 3.2 Neglect of the  dryness and redness of  the skin on the residents’ bony prominences | | | We apply body lotion to the residents after giving them a shower…. We have to do it quickly because we need to finish giving showers to many residents in the morning. We do not focus on specific body parts, but roughly apply the lotion to the whole body, e.g., the chest, abdomen, and limbs. | | - Dry or peeling skin was found on the residents’ ankles, heels, feet, elbows, and back.    - The care staff members applied body lotion roughly to the residents’ chest and abdomen, but they did not apply it to their elbows, knees, ankles, and heels.  - Dry or red skin on the bony prominences were seen in some residents. | |  |
|  |  | | |  | | |  | |  | |  |
| 4 | Improper pressure ulcer prevention practices | | | 4.1 Inappropriate positioning of residents for decreasing the pressure on the sacral areas of the residents | | |  | | - The heads of some beds were  elevated to more than 30 degrees.  - The residents were in a lateral position of either more than 30 degrees or less than 10 degrees. | |  |
|  |  | | | 4.2 The use of inner and outer napkins to reduce workloads | | | In addition to the outer napkin, two inner napkins are wrapped on residents with incontinence. In male residents, a small napkin is also wrapped around the penis…. We only have to pull out the wet inner napkin and do not need to change the outer napkin until it too is wet…. This reduces the need to frequently change napkins and therefore decreases our workload. (PCWs and HWs) | |  | |  |
|  |  | | | 4.3 Method and frequency of changing napkins ineffective at preventing the buttocks  of residents from becoming wet | | | We change napkins two times every shift as scheduled, around once every 3 hours…. We pull out the wet inner napkin and do not need to change the outer napkin until it too is wet. (PCWs and HWs) | | - The buttocks and inner napkins of some residents were wet. | |  |
|  |  | | | 4.4 Use of air-ring cushions or non-pressure relieving cushions. | | | Family members brought air-ring cushions or common soft cushions to chair-bound  residents to serve as seat cushions. Some chair-bound residents sit out in chairs with cushions that were brought by their family members for comfort only, instead of for pressure relief.… I do not see the difference between these two types of  seat cushions…. I also have not seen referrals from doctors or other health professionals to purchase these pressure-relieving seat cushions.” (PCWs and HWs) | | - Air-ring seat cushions and non-  pressure relieving seat cushions were  used instead of pressure relieving seat  cushions for residents sitting in  wheelchairs or chairs. | |  |
|  |  | | | 4.5 Neglecting to fix heel protectors that had slipped off. | | |  | | - Some heel protectors had  slipped off the residents’ legs, but  were not fixed by staff even though  they noticed. | |  |
|  |  | | |  | | |  | |  | |  |
|  |  | | | 4.5 Tight restraint applied to residents | | |  | | - Safety vests and hand restrainers to restrict the body and limb movements of the residents were applied tightly, causing redness over the wrists of some residents and limiting their movements. | |  |
| 5 | Perception that  the preventive care being performed correctly | | | 5.1 Perception of the appropriate length of time for sitting out the chair-bound residents. | | | Normally, the chair-bound residents sit up in the morning for around 3 hours and then again in the afternoon for 2 more hours…. It is not long indeed…. We move them back to bed after every two to three hours. (PCWs) | | - The residents were transferred from their beds to the chairs in the dining rooms after showering at around 9 to 9:30 am and returned to the beds after their lunch at around 12 noon to 12:30 pm. | |  |
|  |  | | | 5.2 Use of inner and  outer napkins to reduce workloads. | | | In addition to the outer napkin, two inner napkins are wrapped around residents with incontinence. In male residents, a small napkin is also wrapped around the penis…. We only have to pull out the wet inner napkin and do not need to change the outer napkin until it too is wet…. This reduces the frequency with which we need to change napkins and therefore decreases our workload.” (PCWs ) | | - The buttocks and inner napkins of the residents were wet. | |  |
| 6. | Inadequate readiness to adopt an assessment tool to assess the risk of developing PUs | | |  | | | We do not think that it is necessary to use the risk assessment tool to assess the risk of developing pressure ulcers…. Those who are bed-ridden and chair-bound develop pressure ulcers more easily we know this…. It is too time-consuming to use the tool. (HWs and nurses) | |  | |  |
|  |  | | |  | | |  | |  | |  |
| 7 | Undesirable environment | | | 7.1 Location of the bed not permitting two care staff members to work together on two different sides of the bed | | | You (the researcher) also know that one side of most beds in the home is in contact with a wall or partition due to the lack of space here…. It is difficult for two of us to work on two sides of the bed to lift and transfer a resident, provide perineal care, and/or change their position, etc…. Such care is usually performed by one staff member on one side of the bed, so it is hard to perform the care well. (PCWs and HWs) | | - Only one member of care staff performed personal care for residents, for example, changing their position and lifting heavy and frail residents onto the bed. | |  |
|  |  | | | 7.2 Many items placed on the beds of residents | | | Many items are put on their beds due to the  limited space for each resident in the home.…  For some residents, their belongings even  occupy half of the bed. It is hard to perform  some procedures, for example changing  the position of the resident.” (PCWs, HWs) | | - There were many items on some beds, such as napkins, extra pillows, and unused heel protectors. This made it difficult for staff to change the position of the residents and to limit their movements. | |  |
| 8 | The supplying of unfavorable resources | | | 8.1. Insufficient pressure ulcer prevention materials supplied to needy residents | | | The PU prevention materials, e.g. pressure-relieving mattresses and heel protectors, are bought by the family members of the residents…. If our home has spare materials, we rent them for use. However, some family members refuse to buy the materials if they do not see PUs on the residents…. Residents who the comprehensive social security allowance can be referred to doctors to be financially supported by the government to purchase the materials,….. but some doctors will not make a referral if the resident has not developed pressure ulcers…. Some residents who are at a high risk of developing pressure ulcers do not have these materials to use. (PCWs, HWs, and nurses) | |  | |  |
|  |  | | | 8.2 No pressure-relieving seat cushions provided to chair-bound residents | | | The family member brought an air-ring cushion to the chair-bound resident as a seat cushion. It was very effective, and the pressure ulcer on the buttocks is getting better now…. Some chair-bound residents sit out in chairs with cushions that were brought by their family members for comfort only, instead of for pressure relief.… I do not see the difference between these two types of seat cushions…. I also have not seen referrals from doctors or other health professionals to purchase these pressure-relieving seat cushions.” (PCWs and HWs) | | - Air-ring seat cushions and non-  pressure relieving seat cushions were  used instead of pressure relieving seat  cushions for residents sitting in  wheelchairs or chairs.  - No pressure-relieving seat cushions were available. | |  |
|  |  | | | 8.3 Insufficient manpower causing PU prevention care to be accorded a lower priority. | | | If we are busy, for example due to a colleague’s sick leave or resignation, we likely will not change the residents’ position and their wet  napkins and sit them up as scheduled. Such things are not a priority. (PCWs) | |  | |  |
|  |  | | |  | | |  | |  | |  |
| 9 | Various management styles in the homes | | 9.1 Supervisory role performed differently in homes with or without nurses | | HWs facing barriers to the active performance of their supervisory role in homes without nurses.  Nurses actively performing their supervisory role to improve the quality of the care provided. | | We are also busy and do not have sufficient time to monitor the work of the PCWs…. We have been colleagues for many years so it is embarssing to tell them what they need to improve. We just gently remind them.… Also, some of them do not follow our instructions and argue with us…. (HWs in the homes without nurses)  The in-charge of this home is an HW … but we observe and monitor the care provided by PCWs and HWs. If they, especially PCWs, do not do well, we remind and instruct them on how to carry out the task…. They sometimes do not follow our instructions, so frequent reminders and teaching are necessary. (Nurses) | |  | |  |
|  |  | 9.2 Different approaches to handing over the cases  in each duty shift. | | | | The cases and required care to be handed over when necessary in the homes without nurses.  The homes with nurses having a formal way to hand over the cases and required care. | |  | | - PCWs were specifically instructed to carry out tasks on some residents when necessary, but there was no formal handing over of cases in each duty shift in the homes without nurses.  - The cases and the tasks that needed to be done were handed over formally in each duty shift in the homes with nurses. | |

Table S2. Changes identified from the focus group interviews and field observations

| 1 |  | | Focus Group Interviews | Field Observations |
| --- | --- | --- | --- | --- |
|  | Themes | Sub-themes | Verbatim | Observation |
|  | Improved compliance with the revised risk assessment method |  | Once every three weeks we are now assessing the risk that chair- and bed-bound residents face in developing PUs.… We can afford the time and frequency for this assessment method. (HWs and nurses) | - The risk factors in the development of PUs for each chair-bound or bed-bound resident were filled in in the modified Braden form. |
| 2 | Timely and  appropriate  use of pressure  ulcer prevention  materials | 2.1 Prevention materials  used by residents without pressure ulcers | Several chair-bound residents with or without pressure ulcers were given a pressure-relieving seat cushion to use. Their family members thanked us for this … and the residents accepted it. As we have only two cushions, we let the residents use them by rotation…. We asked family members to buy them, but they did not respond.” (PCWs and HWs) | - Chair-bound residents with or  without pressure ulcers sat on the seat  cushions by rotation.  - Heel protectors, pressure-relieving  mattresses, and seat cushions were  used by weak and frail residents  without pressure ulcers. |
|  |  | 2.2 Prevention materials used appropriately |  | - Heel protectors were being  applied appropriately, not tightly.  - The pressure ulcer relieving  mattresses were inflated adequately. |
| 3 | Staff empowered to improve the quality of prevention care | 3.1 Inspecting the skin and reporting skin redness and lesions. | Whenever I saw the skin breakdown or skin redness of a resident, I reported it to them (HWs/nurses) for action. I now know that skin redness may be a pressure ulcer at stage one. Previously, we did not manage stage one pressure ulcers until we saw the breakdown, which may be stage two pressure ulcers…. I am not sure whether my identification of a stage one pressure ulcer is correct, but whenever I found skin redness, I reported it to them (HWs/or nurses). (PCW) | - PCWs inspected the residents’ skin while doing perineal care and turning, and they reported any skin redness and skin abrasions of the residents to HWs and/or nurses.  - Stage one pressure ulcers were noted in the files of the residents. |
|  |  | 3.2 Skin kept moistened | We pay more attention to the bony prominences, e.g. the elbows and ankles of the residents, when applying body cream. | - The lower limbs, heels, and elbow areas of many residents were moist.  - Body lotion/creams were placed on the bedside tables of residents who had dry skin for use after perineal care, a change of position, and a shower. |
|  |  | 3.3 More attention and effort paid to the positioning of the residents | We pay more attention to the positioning of residents, making sure that there was less than a 30-degree slope in their lateral position and elevating the bed to a slope of less than 30 degrees to prop up the residents…. Sometimes I forget the proper skills, but my colleagues remind me. (PCWs) | - The majority of residents were positioned in a lateral position with a slope of around 20 to 30 degrees.  - Pillows were being appropriately used to support the majority of the residents in a lateral position to minimize the pressure on the bony prominences. |
|  |  | 3.4 Decreased use of inner and outer napkins for incontinent residents | We now also wrap one inner and one outer napkin around incontinent residents, but only on the night shift. We have fewer staff on the night shift, so it is impossible to change the napkins as often as during the day shift.” (PCWs and HWs) | - One inner napkin and one outer napkin were used for incontinent residents on the night shift only. |
|  |  | 3.5 Increased frequency of changing napkins | We change the napkins according to the schedule, two times in each shift … however, we also check the dryness of the napkins before meals. | - The buttocks and napkins of several residents were still wet. |
|  |  | 3.6 Appropriate use of physical restraints | Physical restraints are the last resort for protecting the residents from harm. We are paying more attention to avoiding the tight application of restraints. | - Safety vests and hand restrainers were appropriately applied to the residents to allow them to reposition themselves on their own in their beds. |
| 4. | Home management | 4.1 Having a formal way to hand over the cases in each duty shift at the homes without nurses. | We now have handover time to report the care issues that have arisen. The HWs tell us more about the residents’ health, including their PU situation and what we should pay more attention to and improve. (PCWs and HWs, the homes without nurses) | - All frontline care staff who were on  duty gathered at the staff counter to  hand over the cases and the care  required in each duty shift at the four  nursing homes. |
|  |  | 4.2 Enhanced communication on care of residents among care staff. | We are now required to briefly report our work before we go off duty. I think this is good, but sometimes I do not know what I should report. I am learning bit by bit. (PCWs in the homes with or without nurses) | - Before they went off duty in the handover time at the four nursing homes, the leads of the PCWs and HWs/nurses in each duty shift briefly reported the care provided to special cases and the care required to start or continue. |
|  |  | 4.3 Improved supervision to PCWs | Many PCWs know the proper prevention practices through the training and cyclical implementation of the protocol…. They are becoming willing to follow our instructions and advice on PU prevention skills, e.g., the positioning of the residents, skin inspections and moisture, the change of napkins…. However, several of them (PCWs) sometimes still argue with us when they are instructed on how to do something better. (HCWs and nurse | - HWs and nurses sometimes reminded PCWs to perform specific care tasks, including fixing heel protectors that have slipped off, changing wet napkins, positioning the residents properly, and the majority of the PCWs performed the tasks accordingly. |
|  | 5. Environment | Decreased clutter on the residents’ beds. | We put the residents’ stuff that had been placed on the beds but were not often used, in their bedside cabinets and also cleared and tidied their beds every day just after the provision of personal care, e.g., changing their position. | - The number of belongings on many residents’ beds was reduced. The majority of the residents could be moved on the bed, either on their own or by care staff. |

**Table S3. The incidence and prevalence of pressure ulcers in the three cycles of implementation**

| Nursing homes | Cycles of implementation | Total number of residents | Number of staff | Resident-  staff ratio (%)  (Number of residents cared for by each care staff member) | Incidence Prevalence | | Number of nurses |
| --- | --- | --- | --- | --- | --- | --- | --- |
| A | Before the protocol implementation | 87 | 19 | 4.58 |  | 5.7% | 3 |
|  | 1 | 85 | 19 | 4.58 | 9.2% | 6.4% |  |
|  | 2 | 94 | 20 | 4.7 | 11.7% | 7.7% |  |
|  | 3 | 91 | 20 | 4.55 | 3.3% | 6.2% |  |
|  |  |  |  |  |  |  |  |
| B | Before the protocol implementation | 175 | 42 | 4.26 |  | 2.8% | 1 (in-charge) |
|  | 1 | 178 | 42 | 4.24 | 3.4% | 5.5% |  |
|  | 2 | 182 | 43 | 4.21 | 4.4% | 2.9% |  |
|  | 3 | 175 | 47 | 3.72 | 1.7% | 2.4% |  |
|  |  |  |  |  |  |  |  |
| C | Before the protocol implementation | 68 | 12 | 5.57 |  | 4.4% |  |
|  | 1 | 69 | 12 | 5.75 | 8.8% | 4.3% | 0 |
|  | 2 | 74 | 10 | 7.4 | 7.2% | 4.1% |  |
|  | 3 | 71 | 10 | 7.1 | 5.4% | 5.4% |  |
|  |  |  |  |  |  |  |  |
| D | Before the protocol implementation | 140 | 25 | 5.6 |  | 4.9% | 0 |
|  | 1 | 142 | 26 | 5.46 | 4.9% | 2.9% |  |
|  | 2 | 137 | 27 | 5.07 | 4.4% | 3.8% |  |
|  | 3 | 132 | 27 | 4.67 | 4.5% | 4.5% |  |
|  |  |  |  |  |  |  |  |
